# Combination of different noninvasive brain stimulation treatments for upper limb recovery in stroke patients: A systematic review

**DOI:** 10.1002/brb3.3370

**Published:** 2024-01-29

**Authors:** Lerín‐Calvo Alfredo, Rodríguez‐Martínez David, Bernal‐Jiménez Juan‐José, Lerma‐Lara Sergio, Ferrer‐Peña Raúl

**Affiliations:** ^1^ Departamento de Fisioterapia, Centro Superior de Estudios Universitarios La Salle Universidad Autónoma de Madrid Aravaca Madrid Spain; ^2^ Clínica Neuron Madrid Río Madrid Spain; ^3^ Grupo de Investigación Clínico‐Docente sobre Ciencias de la Rehabilitación (INDOCLIN) CSEU La Salle, UAM Aravaca Madrid Spain; ^4^ Motion in Brains Research Group Aravaca Madrid Spain; ^5^ Faculty of Health Sciences Universidad de Castilla la Mancha Talavera de la Reina Toledo Spain

**Keywords:** noninvasive brain stimulation, transcranial magnetic stimulation, transcranial direct current stimulation, motor control, upper extremity, neuroscience, stroke

## Abstract

We report a review of Pubmed (Medline), CENTRAL, Web of Science, and Scopus to test the effectiveness of the combined application of repetitive transcranial magnetic stimulation and transcranial direct current stimulation in the improvement of different functional variables of the upper limb in people with stroke. Two independent reviewers assessed eligibility and evaluated the quality of the studies. Five articles were included in the final review according to the inclusion criteria: Most show statistically significant differences in motor function improvement in favor of the experimental group, but not in activity. Due to the heterogeneity of the observed studies, the results should be interpreted with caution—more high‐quality studies are needed to investigate the effectiveness of these interventions in different stages of stroke patients.

## 1 INTRODUCTION

Stroke is one of the leading causes of death and disability globally (Feigin et al., 2014). About 80% of stroke survivors report a loss of upper limb motor function (Faria‐Fortini et al., 2011), which greatly affects their quality of life (Demers & Levin, 2017; Faria‐Fortini et al., 2011; Morris et al., 2013).

Some of the current treatment techniques for upper limb rehabilitation after stroke include task‐oriented training, constraint‐induced movement therapy, or robotic‐assisted therapy (Hussain et al., 2022; Tedla et al., 2022; Wu et al., 2021). All these interventions represent a new scenario in the recovery of motor function because they are based on the various advances observed in recent years in neuroscience, which have shown that the phenomena of adaptive plasticity after a central nervous system (CNS) injury can be favored by including treatment protocols that encourage a large number of repetitions of a movement with a high intensity and within a favorable context for the patient (Maier et al., 2019; Noé et al., 2023).

Following this body of evidence, other techniques have emerged to modulate the CNS and enhance the effect achieved with these therapies, for example, motor imagery, action observation, mirror therapy, and noninvasive brain stimulation techniques (NIBS) (Ahmed et al., 2023; Borges et al., 2018; Monteiro et al., 2021; Thieme et al., 2018). The underlying neurophysiological assumptions of these techniques are based on activating the areas related to movement planning, which will be activated later during the therapies such as the dorsolateral prefrontal cortex, the supplementary motor area, or the primary motor area; the aim is to increase the excitability of the neurons involved in the performance of the target movements to improve their effectiveness (Cuenca‐Martínez et al., 2020; Koch & Caltagirone, 2020; Maier et al., 2019).

Among the different forms of NIBS, there are two main modalities: repetitive transcranial magnetic stimulation (rTMS) and transcranial direct current electrical stimulation (tDCS). rTMS involves the application of a magnetic current through copper coils that generates an electrical current capable of acting on interneurons located in the cerebral cortex (Masahiro & Wataru, 2014). rTMS can be applied in an excitatory way using frequencies higher than 5 Hz to increase the excitability of neurons located in the desired area. Alternatively, it can be used in an inhibitory way with frequencies of 1 Hz or lower with the aim of promoting inhibition of neuronal activity in the area to be stimulated (Klomjai et al., 2015).

tDCS is based on similar neurophysiological principles to rTMS and involves passing a weak current (approximately 2 mA) to the cerebral cortex using electrodes placed directly on the scalp (Fritsch et al., 2010). The direction of the electrical current is routed from the anode to the cathode so that the anode is placed in the part of the cortex where neuronal excitability is intended to be increased. The cathode is placed in the area that is intended to be inhibited (Lefebvre & Liew, 2017).

The most commonly used set‐ups in clinical practice for the application of tDCS in upper limb rehabilitation are anode placement in the primary motor cortex (M1) of the affected hemisphere and cathode placement in M1 of the healthy hemisphere (Lindenberg et al., 2010), placement of the anode in M1 of the affected hemisphere and the cathode in the supraorbital area of the contralateral side, and the placement of the cathode in M1 of the healthy hemisphere and the anode in the supraorbital area of the contralateral side (Hesse et al., 2011). Both rTMS and tDCS have been shown to be effective in improving motor function and upper limb motor control in people with stroke (Chen et al., 2022; Tang et al., 2022); however, the use of both techniques simultaneously or contiguously has not been demonstrated in the literature.

Therefore, the aim of this systematic review was to determine the effects of the combination of rTMS and tDCS on the recovery of motor function and improvement of upper limb motor control in stroke patients.

## 2 METHODS

This systematic review was conducted according to the PRISMA 2020 (preferred reporting items for systematic reviews and meta‐analysis) statement that was recently updated (Page et al., 2021). This review was registered in PROSPERO (ID: CRD42023417949).

### 2.1 Inclusion criteria

The selection criteria used in this review were based on clinical and methodological aspects such as population, intervention, control, outcomes, and study design. The criteria were thus based on trying to answer the PICO question (population (P); intervention (I); control (C); and dependent variable(s) or result(s) of interest (O)) (Stone, 2002).

#### 2.1.1 Population

Randomized clinical trials (RCTs) were used in which an experimental intervention group was compared to a control group. No language or time filters were applied. Patients in the studies had to have suffered a stroke and have motor impairment in the upper limb.

#### 2.1.2 Intervention and control treatment

The selected studies were RCTs in which the main intervention was based on the combined use of rTMS and tDCS (either applying rTMS and tDCS simultaneously or implementing one technique immediately after the other) or the use of rTMS followed by tDCS. The control groups received either rTMS alone or rTMS placebo or real rTMS with tDCS placebo.

#### 2.1.3 Results

Variables studied to test the results and the effect of the treatment were motor function and motor control. To assess motor function, we used the Fugl‐Meyer scale in its upper limb version. To assess motor control, we used tests such as the wolf motor function test (WMFT), the action research arm test (ARAT), and various motor control tests such as bimanual coordination, gripper strength, or accuracy/speed in performing a motor task.

### 2.2 Search strategy

Two independent reviewers searched scientific articles, thus generating an agreement for the initial selection of studies after which discordances were sought. The search for scientific articles was conducted using the databases Pubmed, Cochrane Central Register of Controlled Trials (CENTRAL), Web of Science, and EMBASE. The terms used for the search strategy were as follows: “rTMS,” “tDCS,” “stroke,” “cerebrovascular accident,” “motor function,” “motor deficits,” “motor control,” “upper limb,” “hand,” “arm,” and “upper extremity.” Phase one of the search for scientific articles ended in May 2023. The search strategy used for each of the databases is reflected in the Appendix.

### 2.3 Selection criteria and data extraction

An analysis of the information was first carried out by two independent reviewers who assessed the relevance of the RCTs with respect to the research objective. The studies included should examine the effectiveness of the combined use of rTMS and tDCS (either applying rTMS and tDCS simultaneously or implementing one technique immediately after the other) in upper limb motor function of stroke patients by measuring changes observed in scales like Fugl‐Meyer, ARAT, WMFT, or specific motor tasks that evaluate motor control. The first analysis was performed based on the information extracted from the title and abstract of the studies. If there was no consensus or the abstracts did not contain the necessary information, the full text was accessed.

**FIGURE 1:**
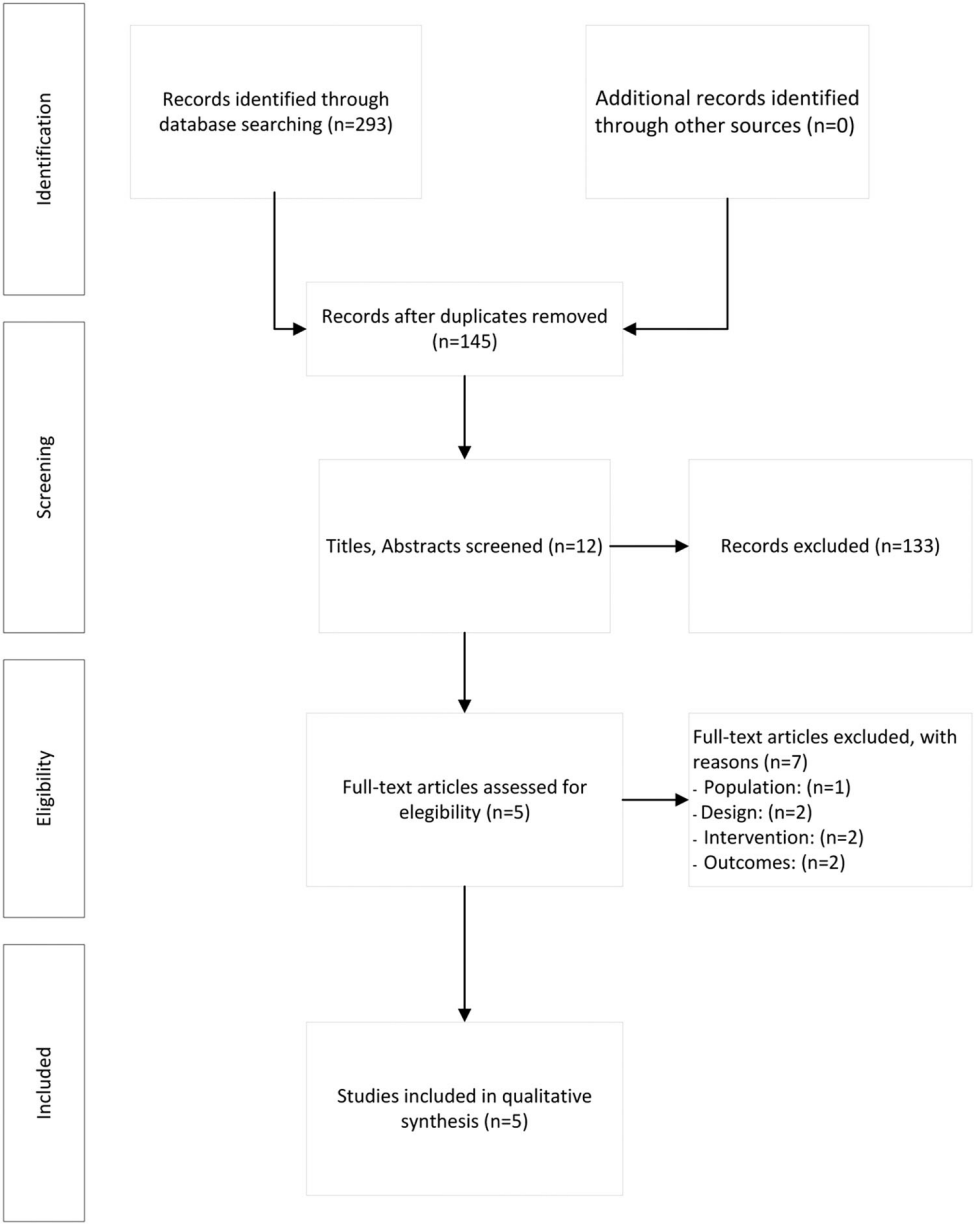
Study search strategy flowchart (preferred reporting items for systematic reviews and meta‐analysis [PRISMA]).

In the second phase of the analysis, a full‐text reading of the articles was performed to check which articles met the inclusion criteria. Differences between reviewers were resolved by consensus by a third reviewer. The flowchart related to the selection criteria is detailed in Figure 1.

### 2.4 Risk of bias assessment

Two authors independently assessed the risk of bias of each study, using the Cochrane risk of bias scale (version 5.1.0). The scale comprised seven domains: selection bias (random sequence generation and allocation concealment); conduct bias (blinding of participants and staff); detection bias (blinding of outcome assessor); attrition bias (incomplete outcome data); reporting bias (selection of reported outcomes); and other potential sources of bias. Each domain was scored as yes, no, and unclear and classified into one of three categories: high risk of bias, low risk of bias, or unclear.

Two independent reviewers examined the quality of all selected studies using the same methodology. Disagreements between reviewers were resolved by consensus with a third reviewer. Inter‐rater agreement (inter‐rater reliability) was measured using Cohen's kappa coefficient (*κ*) as follows: (1) *κ* > .7 indicates a high level of inter‐rater agreement; (2) *κ* = .5–.7 indicates a moderate level of agreement; and (3) *κ* < .5 indicates a low level of agreement (Landis & Koch, 1977).

### 2.5 Methodological quality assessment

The methodological quality of the studies was assessed using the PEDro scale (Maher et al., 2003), which consists of 11 criteria to assess the internal and external validity of the studies. The 11 criteria assessed by the scale are as follows: (1) specified study eligibility criteria; (2) random allocation of patients; (3) concealed allocation; (4) measure of similarity between groups at baseline; (5) patient blinding; (6) therapist blinding; (7) assessor blinding; (8) fewer than 15% dropouts; (9) intention‐to‐treat analysis; (10) intergroup statistical comparisons; and (11) point measures and variability data. Methodological criteria were scored as follows: yes (1 point), no (0 points), or do not know (0 points). The PEDro score for each selected study provided an indicator of methodological quality (9–10 = excellent; 6–8 = good; 4–5 = fair; 3–0 = poor) (Hariohm et al., 2015).

To identify risk of bias, two independent reviewers examined the quality of all selected studies using the same methodology. Disagreements between reviewers were resolved by consensus with a third reviewer. Inter‐rater agreement (inter‐rater reliability) was measured using Cohen's kappa coefficient (*κ*) as follows: (1) *κ* > .7 indicates a high level of inter‐rater agreement; (2) *κ* = .5–0.7 indicates a moderate level of agreement; and (3) *κ* < .5 indicates a low level of agreement (Landis & Koch, 1977).

### 2.6 Qualitative analysis

To evaluate qualitative analysis of the results, the evidence was classified in five grades depending on the methodological quality of the studies as follows (van Tulder et al., 2003):

1. Strong evidence: Provided by statistically significant findings on outcome measures in at least two high quality RCTs.
2. Moderate evidence: Provided by statistically significant findings on outcome measures in at least one high quality RCT and at least one low quality RCT and/or one high quality controlled clinical trial (CCT).
3. Limited evidence: Provided by statistically significant findings on outcome measures in at least one high‐quality RCT and/or at least two high‐quality CCTs (in the absence of a high‐quality RCT).
4. Indicative findings: Provided by statistically significant findings on outcome measures in at least one high quality CCT and/or low quality RCT (in the absence of high quality RCTs) and/or two studies of a nonexperimental nature of sufficient quality (in the absence of RCTs and CCTs).
5. No or insufficient evidence: Cases where the results of eligible studies did not meet the criteria for one of the levels of evidence indicated above and/or in the case of conflicting results (statistically significant positive and statistically significant negative) between RCTs and CCTs, or in the case of a lack of eligible studies.

## 3 RESULTS

Figure 1 shows a flowchart of the study. Of the 293 studies, 5 met the study inclusion criteria and were included in this review. Table 1 shows the characteristics of the studies with respect to sample size, population, intervention, and measured variables.

**TABLE 1:** Demographic data and protocols of studies.

Reference	Treatment	Number of patients (M/F)	Age (mean ± SD)	Months/week/days poststroke (mean ± SD)	Type of stroke (I/H)	Affected hemisphere (L/R)	Therapy protocol	Outcome measures	Results
Kwon et al. (2016)	I1: rTMS + tDCS	20 (14/6)	58.6 ± 8.4	42.0 ± 37.5 months	17/3	11/9	I1: 1000 pulses, 100 trains 10 Hz rTMS 90% RMT over ipsilesional hemisphere + cathodal tDCS contralesional hemisphere	Accuracy in a motor task; time in a motor task; MEP amplitude; MEP latency	Statistically significant differences between groups in time required for the motor task in favor of I1 but no differences between groups in accuracy
Crossover	I2: rTMS + tDCS						I2: 1000 pulses, 100 trains 10 Hz rTMS 90% RMT over ipsilesional hemisphere + anodal tDCS contralesional hemisphere		
	I3: tDCS and rTMS						I3: Cathodal tDCS contralesional hemisphere (10 min) and 1000 pulses, 100 trains 10 Hz rTMS 90% RMT over ipsilesional hemisphere (10 min) (after tDCS application)		
	I4: tDCS and rTMS						I4: Anodal tDCS contralesional hemisphere (10 min) tDCS cathodal and 1000 pulses, 100 trains 10 Hz rTMS 90% RMT over ipsilesional hemisphere (10 min) (after tDCS application)		
	C: Sham tDCS + rTMS						C: Sham tDCS contralesional hemisphere + 1000 pulses, 100 trains 10 Hz rTMS 90% RMT over ipsilesional hemisphere		
Pipatsrisawat et al. (2022)	I: rTMS and tDCS	I: 5 (1/4)	I: 58.8 ± 5.9	I: 35.2 ± 21.7 days	I: 4/1	I: 3/2	I: 1200 pulses, 1 Hz rTMS 100% RMT contralesional hemisphere and cathodal tDCS contralesional hemisphere	FMA‐UE; WMFT‐FA; WMFT‐time	Statistically significant improvement in FMA‐UE for the intervention group but no differences in WMFT‐FA or WMFT‐time
	C: Sham rTMS or sham tDCS	C: 5 (1/4)	C: 59.2 ± 17.5	C: 47.4 ± 25 days	C: 4/1	C: 3/2			
Cho et al. (2017)	I: rTMS + tDCS	I: 15 (9/6)	I: 60.73 ± 9.28	I: 13.8 ± 6.2 months	I: 12/3	I: 7/8	I: 1000 pulses, 10 Hz rTMS 90% RMT ipsilesional hemisphere + cathodal tDCS contralesional hemisphere	FMA‐UE; FMA‐LE; FMA T; MEP amplitude; MEP latency	Statistically significant improvement in FMA‐UE and FMA‐T for the intervention group
	C: rTMS	C: 15 (8/7)	C: 58.13 ± 12.52	C: 13.6 ± 5.2 months	C: 13/2	C: 7/8	C: 1000 pulses, 10 Hz rTMS 90% RMT ipsilesional hemisphere		
Takeuchi et al. (2012)	I: rTMS + tDCS	I: 9 (6/3)	I: 57 ± 10.02	I:62 ± 33.2 months	NR	I: 6/3	I: 1200 pulses, 1 Hz rTMS 90% RMT contralesional hemisphere + anodal tDCS ipsilesional hemisphere	Bimanual coordination; pinch force; MEP amplitude; TCI	Statistically significant changes in Pinch force for the intervention group and C1 group, but no differences between them.
	C1: rTMS + sham tDCS	C1: 9 (6/3)	C1: 64 ± 5.8	C1: 71.9 ± 51 months		C1: 3/6	C1: 1200 pulses, 1 Hz rTMS 90% RMT contralesional hemisphere + sham tDCS ipsilesional hemisphere		No differences in bimanual coordination between groups
	C2: tDCS + sham rTMS	C2: 9 (5/4)	C2: 63.4 ± 6.7	C3:67.4 ± 61 months		C2: 4/5	C2: anodal tDCS ipsilesional hemisphere + sham rTMS contralesional hemisphere		
Lee et al. (2018)	I: rTMS + tDCS	I:12 (8/4)	I: 56 ± 13.4	I:20 ± 8.7 days	I:7/5	I:7/5	I: 1000 pulses, 10 Hz rTMS 90% RMT ipsilesional hemisphere + cathodal tDCS contralesional hemisphere	FMA; Interhemispheric connectivity	No statistically significant differences between groups for FMA
	C: rTMS + sham tDCS	C: 12(9/3)	C: 54.8 ± 15.5	C: 15.5 ± 5.3 days	C:11/1	C:5/7	C: 1000 pulses, 10 Hz rTMS 90% RMT ipsilesional hemisphere		

*Note*: “+”: Simultaneous application of both techniques; “and”: application of one technique followed by the other.

Abbreviations: C, Control Group; FMA‐LE, Fugl‐Meyer Assessment‐lower extremity; FMA‐T, Fugl‐Meyer Assessment‐total; FMA‐UE, Fugl‐Meyer Assessment‐upper extremity; Hz, hertz; I, intervention group; MEP, motor evoked potentials; RMT, resting motor threshold; rTMS, repetitive transcranial magnetic stimulation; TCI, transcallosal inhibition; tDCS, transcranial current direct stimulation; WMFT‐FA, wolf motor function test‐functional ability; WMFT‐Time, wolf motor function test‐performance time.

### 3.1 Characteristics of the study population

All patients included in the studies suffered a stroke with upper limb limitation. The type of design used for the search was randomized controlled trials, and the study samples varied. The total number of patients included was 111.

### 3.2 Characteristics of the interventions

All studies applied interventions based on the combination of rTMS and tDCS, which could be used simultaneously or by applying one followed by the other. None of the evaluated studies combined noninvasive stimulation techniques with any other technique. A detailed description of the interventions is given in Table 2.

**TABLE 2:** Stimulation parameters of studies.

Reference	Participants, phase poststroke	NIBS					Type of control group
		NIBS paradigm	Timing of stimulation	Intensity of frequency of current	Number of sessions	Number of weeks	
Kwon et al. (2016)	3 subacute 17 chronic	Simultaneous HF‐rTMS (ipsilesional) and ctDCS (contralesional) vs. simultaneous HF‐rTMS (ipsilesional) and atDCS (contralesional) vs. ctDCS contralesional before HF‐rTMS ipsilesional vs. atDCS contralesional before HF‐rTMS ipsilesional vs. sham tDCS contralesional + rTMS ipsilesional	Simultaneous 20 min vs. 10 min tDCS and 20 min of rTMS	10 Hz and 2 mA	1	1	Sham tDCS + real rTMS
Pipatsrisawat et al. (2022)	Subacute	LF‐rTMS contralesional y ctDCS contralesional	20 min rTMS and 20 min tDCS	1 Hz and 2 mA	1	1	Sham tDCS or sham rTMS
Cho et al. (2017)	Chronic	Simultaneous HF‐rTMS ipsilesional + ctDCS contralesional	20 min	10 Hz and 2 mA	10	2	Real rTMS
Takeuchi et al. (2012)	Chronic	Simultaneous LF‐rTMS contralesional + atDCS ipsilesional	20 min	1 Hz and 1 mA	1	1	Real rTMS + sham tDCS
							Real tDCS + sham rTMS
Lee et al. (2018)	Subacute	Simultaneous HF‐rTMS ipsilesional + ctDCS contralesional	20 min	10 Hz and mA	10	2	Real rTMS

Abbreviations: aTDCS, anodal transcranial direct current stimulation; ctDCS, cathodal transcranial direct current stimulation; HF‐rTMS, high frequency‐repetitive transcranial magnetic stimulation; Hz, hertz; LF‐rTMS, low frequency‐repetitive transcranial magnetic stimulation; mA, milliamperes; rTMS, repetitive transcranial magnetic stimulation; tDCS, transcranial direct current stimulation.

### 3.3 Methodological quality and risk of bias results

The methodological quality of the studies was assessed with the PEDro scale, whereby four of the studies showed good methodological quality, with a score between 6 and 8, whereas the remaining study showed moderate methodological quality. Table 3 shows the results obtained from the assessment of the studies according to the PEDro scale. The two reviewers reported discrepancies in the assessment of two RCTs; the discrepancy was observed in two studies for items 5 and 7 (4: equality between the groups at baseline; 7: blinding of the assessors). Conflicts were resolved by consensus through a third reviewer. The inter‐rater reliability of the methodological quality assessment was high (*κ* = .927).

**TABLE 3:** Assessment of the studies quality based on PEDro scale.

Reference	1	2	3	4	5	6	7	8	9	10	11	Total
Kwon et al. (2016)	0	1	0	1	1	0	0	1	1	1	0	6
Pipatsrisawat et al. (2022)	1	1	1	1	1	0	0	1	1	1	0	7
Cho et al. (2017)	0	1	0	1	0	0	0	1	1	1	0	5
Takeuchi et al. (2012)	0	1	0	1	1	0	0	1	1	1	0	6
Lee et al. (2018)	0	1	0	1	0	0	1	1	1	1	0	6

*Note*: (1) Subject choice criteria are specified; (2) random assignment of subjects to groups; (3) hidden assignment; (4) groups were similar at baseline; (5) all subjects were blinded; (6) all therapists were blinded; (7) all evaluators were blinded; (8) measures of at least one of the key outcomes were obtained from more than 85% of baseline subjects; (9) intention‐to‐treat analysis was performed; (10) results from statistical comparisons between groups were reported for at least one key outcome; and (11) the study provides point and variability measures for at least one key outcome.

**FIGURE 2:**
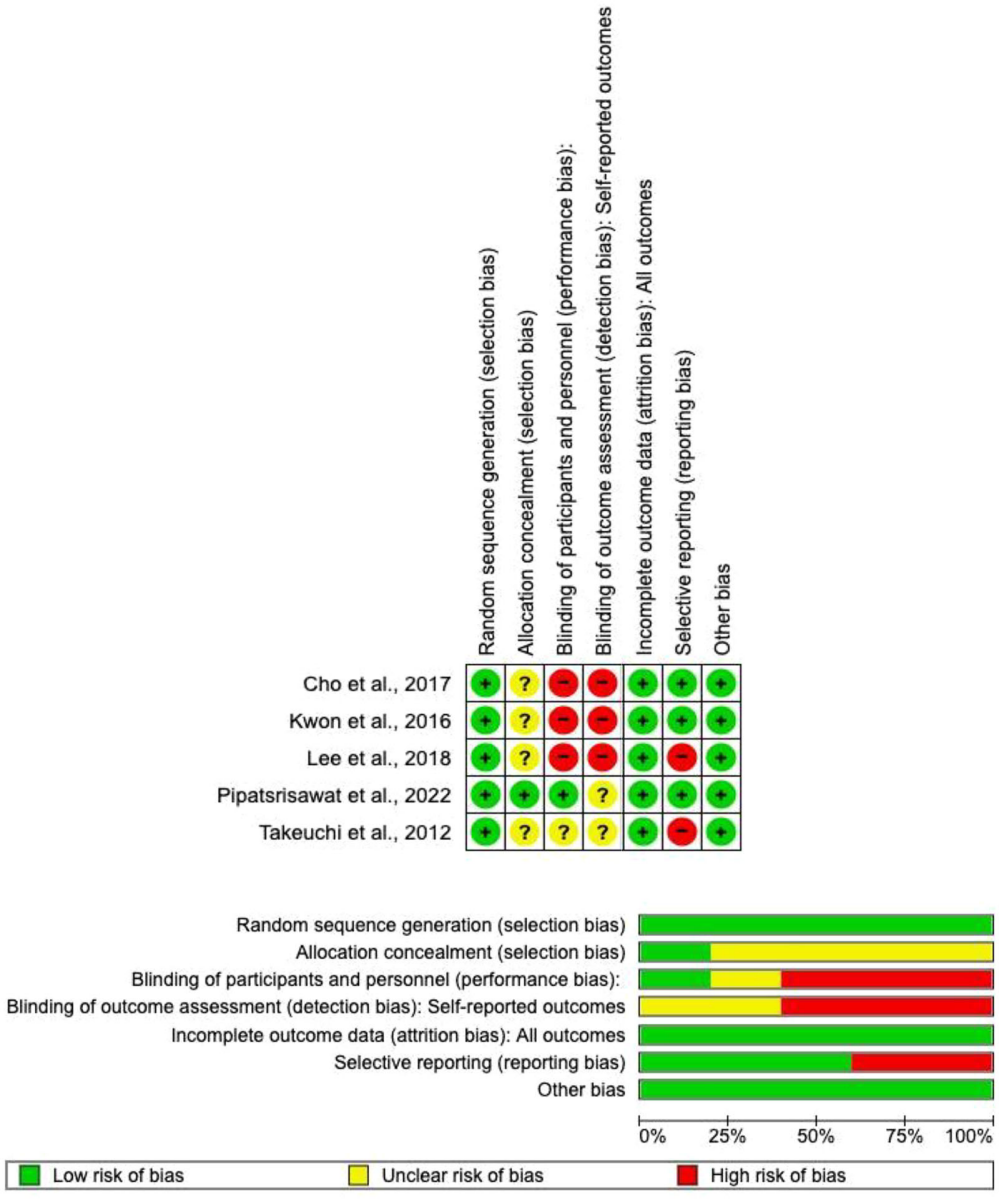
Risk of bias summary: (+) low risk of bias; (?) uncertain risk of bias; (—) high risk of bias.

Risk of bias of the studies was assessed with the Cochrane risk of bias scale (version 5.1.0) with most of them showing a low risk of attrition bias. The attrition bias domain had highest percentage of studies with a high risk of bias. Figure 2 shows the summary of the risk of bias and the risk of bias graph. The inter‐rater reliability of the Cochrane risk of bias scale was good (*κ* = .740).

### 3.4 Comparisons of the interventions

#### 3.4.1 Combination of rTMs and tDCS versus sham rTMS and/or tDCS

There was only one study that compared the effectiveness of rTMS in combination with tDCS compared to a placebo group. The Pipatsrisawat group compared the application of low‐frequency rTMS applied over the contralesional hemisphere followed by cathodal tDCS over the contralesional hemisphere with placebo application of rTMS and tDCS. After stimulation, differences were observed between groups in motor function assessed with the Fugl‐Meyer Assessment (FMA)‐UL 1 week after stimulation. However, despite finding improvements over time in both groups on the WMFT scale—and a trend of improvement in the experimental group—no statistically significant differences were observed for this variable between the two groups.

#### 3.4.2 Combination of rTMs and tDCS versus rTMS or tDCS

Among the observed studies, four compared the simultaneous application of rTMS and tDCS with real stimulation with rTMS including three of them with placebo stimulation with tDCS (Kwon et al., 2016; Lee et al., 2018; Takeuchi et al., 2012); the other one compared it with the application of rTMS in isolation without placebo (Cho et al., 2017). Furthermore, the only study that compared the experimental group with a real tDCS and rTMS placebo group was the study by Takeuchi.

Thus, Cho et al. observed statistically significant changes between the two groups for motor function assessed with the FMA‐UE after 2 weeks of treatment, which were maintained 2 months after the end of treatment. Lee et al. showed no significant differences between the two groups after 2 weeks of stimulation despite observing a greater tendency toward improvement for the same variable in favor of the experimental group.

On the other hand, Kwon showed significant improvements favoring the group that applied simultaneous rTMS stimulation in the injured hemisphere with tDCS in the healthy hemisphere in the motor control for the execution speed variable, but not for movement precision. In contrast, Takeuchi's group observed changes in manual coordination after the application of a stimulation session favorable to the rTMS stimulation group; this was not observed in the rTMS and tDCS stimulation group or in the tDCS stimulation group.

### 3.5 Qualitative analysis

There is limited evidence for the application of rTMS and tDCS simultaneously for the improvement of upper limb motor function in stroke patients (Cho et al., 2017; Lee et al., 2018; Pipatsrisawat et al., 2022).

There is, however, insufficient evidence to support the improvement of specific aspects of motor control such as execution time or gripper strength following the combination of rTMS and tDCS compared to the application of rTMS (Kwon et al., 2016; Pipatsrisawat et al., 2022; Takeuchi et al., 2012).

## 4 DISCUSSION

The aim of this systematic review was to analyze the effectiveness of the combination of rTMS and tDCS in improving upper limb motor function in stroke patients. The results show a trend toward an improvement in upper limb motor ability in these patients measured with the FMA scale, but there is no evidence at the moment of an improvement in motor function measured with skill acquisition tasks or scales such as the WMFT.

This review article considers the International Classification of Functionality (ICF) to distinguish improvements in body structures and functions from improvements in activity (Vargus‐Adams & Majnemer, 2014). Hence, FMA explores changes obtained in body structures, whereas changes obtained in WMFT or in specific skill acquisition tasks are referred to as activity (Madroñero‐Miguel & Cuesta‐García, 2023). The main difference between these two concepts is that the activity is directed toward the performance of a behavioral task, in which accuracy and execution time for the performance of a movement are considered (Shumway‐Cook, 2011; Winstein et al., 2014).

Lee's study showed that the simultaneous application of rTMS and tDCS can increase the intra‐hemispheric connectivity of the affected hemisphere and decrease the intra‐hemispheric connectivity of the healthy hemisphere significantly with respect to the group that applied rTMS alone (Lee et al., 2018). As NIBS began to be applied under the hypothesis of improving interhemispheric imbalance (Williams et al., 2010), the use of these two techniques simultaneously or one before the other could represent an opportunity to increase their efficacy based on their underlying neurophysiological principles. It would suppose an increase in the corticospinal pathway excitability, as seen in Kwon et al. (2016) and in motor capacity of the hemiparetic upper limb in stroke patients (Saini et al., 2022).

Among the protocols used for the application of rTMS, we can identify inhibitory stimulation (low frequency‐repetitive rTMS [LF‐rTMS]) applied directly to the healthy hemisphere of the patient and excitatory stimulation (high frequency‐repetitive rTMS [HF‐rTMS]) applied to the affected hemisphere. Both, when applied in isolation, can improve upper limb motor function after stroke (Rossi et al., 2021). However, several studies have recently been performed to evaluate the possibility of applying both techniques one after the other (Chen et al., 2021). We found studies reflecting a greater effectiveness of HF‐rTMS than LF‐rTMS for the improvement of upper limb motor function (Xue et al., 2022). However, we also found a recent meta‐analysis suggesting that bilateral stimulation may be more effective in patients in the acute phase of stroke than single hemisphere stimulation when comparing the effectiveness according to the time of evolution of the patient. Nevertheless, this does not seem to report better results in patients in the subacute or chronic phase (Chen et al., 2022).

The results of our review are similar to those reported by Ahmed who published a meta‐analysis of improvements in motor function measured with the FMA in stroke patients after applying different types of stimulation protocols with rTMS. Ahmed found no significant changes in comparison with the placebo group when the studies assessed activity with scales such as the WMFT (Ahmed et al., 2023).

tDCS can also be applied with various set‐ups including anodal stimulation (a‐tDCS), cathodal stimulation (c‐tDCS), and dual stimulation (d‐tDCS; the most common) (List et al., 2015; Vines et al., 2008). Recent studies have shown inconclusive results when applying tDCS for motor skill improvement using scales such as the FMA (Chow et al., 2022); however, considering the ICF division, that same study and other systematic reviews with meta‐analyses have shown that all types of tDCS stimulation are effective, but it appears that ctDCS may have greater effectiveness in improving activities of daily living and independence (Chow et al., 2022; Elsner et al., 2020, 2017). The atDCS approach appears to show more significant improvements in motor ability (Xue et al., 2022).

The variability observed in different studies could justify the lack of evidence of activity improvement observed in the current review because some papers observed changes in activity when using HF‐rTMS with c‐TDCS (Kwon et al., 2016), whereas no such improvements were observed for the same variable when using a protocol of LF‐rTMS and a‐tDCS.

## 5 LIMITATIONS

There are many limitations that must be considered when interpreting the study because the results obtained in the review show a great heterogeneity in the studies found in the current scientific literature. Due to the great difference between the application times of the therapies—as well as the different types of set‐ups used, and the variables considered—it is not possible to draw strong conclusions about the effectiveness of the combination of both therapies for the improvement of upper limb motor function in stroke patients. This is why it was not possible to perform a meta‐analysis that could provide more precise information when comparing the different interventions with respect to the main variable. Furthermore, among the studies analyzed, there are several that only use one treatment session; therefore, it is possible that several sessions may be necessary to observe significant changes in these patients. In fact, some studies present methodological errors and small sample sizes that can lead to considerable biases in the interpretation of the results.

## 6 FUTURE RESEARCH LINES AND CLINICAL APPLICATION OF RESULTS

The initial results of the review seem to indicate a greater effectiveness of the combination of rTMS and tDCS compared to the use of one of the two techniques in isolation for improving upper limb motor function in stroke patients; however, it is necessary to carry out studies with high methodological quality in which a higher dose of treatment is applied (from approximately 20 sessions; Chen et al., 2022) to observe significant changes in these patients as well as to understand the changes that the application of these techniques can bring about in the short, medium, and long term. Further studies are needed to understand what type of protocols may be most effective according to the patient.

This review does provide a great advance with respect to the treatment of these patients despite not finding strong evidence of the combination of both techniques. The risk–benefit ratio when applied at the same time is greater than the application of a single therapy. The time invested during the dual session is the same as a single session, and thus, the impact can be improved.

## 7 CONCLUSIONS

The combination of rTMS and tDCS appears to be more effective in improving upper limb motor function in stroke patients than using one of the two techniques in isolation; however, there are no differences when the variable considered is changes in activity.

However, the results should be interpreted with caution due to the heterogeneity of the studies observed. It is not possible to determine the dose or type of protocol that may have the most benefit in these patients. Further high‐quality studies are needed to investigate the effectiveness of these interventions in different stages of stroke patients.

## AUTHOR CONTRIBUTIONS

**Lerín‐Calvo Alfredo**: Conceptualization; investigation; methodology; writing—original draft; writing—review and editing; project administration; formal analysis. **Rodríguez‐Martínez David**: Conceptualization; investigation; methodology; formal analysis. **Bernal‐Jiménez Juan‐José**: Methodology; formal analysis; visualization. **Lerma‐Lara Sergio**: Conceptualization; writing—review and editing; supervision. **Ferrer‐Peña Raúl**: Supervision; writing—review and editing; conceptualization; investigation; methodology.

## CONFLICT OF INTEREST STATEMENT

Authors are required to disclose any potential conflicts of interest that could be perceived as influencing the research or interpretation of results.

### PEER REVIEW

The peer review history for this article is available at https://publons.com/publon/10.1002/brb3.3370.

## PLAGIARISM

We strictly prohibit the submission of any material that constitutes plagiarism or infringes on the intellectual property rights of others.

## DATA INTEGRITY AND FABRICATION

We require that all data presented in submitted manuscripts accurately represent the results of the research. Fabrication or falsification of data is strictly prohibited.

## COMPLIANCE WITH REGULATIONS

All research must comply with relevant national and international regulations governing the conduct of research.

## CORRECTIONS AND RETRACTIONS

In cases of identified errors or misconduct, we will promptly issue corrections or retractions, as appropriate.

## Data Availability

The data that support the findings of this study are openly available in PROSPERO at https://www.crd.york.ac.uk/prospero/, reference number CRD42023417949.

## 1

SEARCH STRATEGY

Pubmed (MEDLINE): 42 trials

(“rTMS” OR “repetitive Transcranial Magnetic Stimulation” OR “TMS” OR “Transcranial Magnetic Stimulation” OR “TBS” OR “theta burst stimulation” OR “Burst stimulation”) AND (“transcranial direct current stimulation” OR “tDCS” OR (“Transcranial Alternating Current Stimulation”) OR (“Transcranial Electrical Stimulation”)) AND (“Stroke” OR “Stroke”[Mesh] OR “Cerebrovascular accident” OR “CVA”) AND (“Motor function” OR “motor deficits” OR “motor control” OR “motor learning” OR “motor”) AND (“upper limb” OR hand OR arm OR “upper extremity”[Mesh])

Cochrane: Cochrane Central Register of Controlled Trials CENTRAL: 31 trials

**Table:**

1	MeSH descriptor: (transcranial magnetic stimulation) explode al trees
2	rTMS OR TMS OR TBS
3	1 OR 2
4	MeSH descriptor: (Transcranial direct current stimulation) explode al trees
5	tDCS or transcranial alternating current stimulation
6	4 OR 5
7	MeSH descriptor: (stroke) explode all trees
8	MeSH descriptor: (motor function) explode all trees
9	Motor function OR motor control OR motor learning
10	8 OR 9
11	MeSH descriptor: (upper extremity) explode all trees
12	Upper limb OR arm OR hand
13	11 OR 12
14	3 AND 6 AND 7 AND 10 AND 13

Web of science: 142 articles

TS = (repetitive transcranial magnetic stimulation OR transcranial magnetic stimulation OR rtms OR tms) AND TS = (transcranial direct current stimulation OR tdcs) AND TS = (stroke OR cerebrovascular disease OR cva) AND TS = (motor function OR motor deficit OR motor control OR motor learning) AND TS = (upper extremity OR upper limb OR arm OR hand)

Embase: 85 trials

(rTMS OR repetitive transcranial magnetic stimulation OR tms OR transcranial magnetic stimulation OR tbs OR theta burst stimulation OR burst stimulation) AND (transcranial direct current stimulation OR tdcs OR transcranial alternating current stimulation OR transcranial electrical stimulation) AND (stroke OR stroke/exp OR cerebrovascular accident OR cva) AND (motor function OR motor deficits OR motor control OR motor learning OR motor) AND (upper limb OR hand OR arm OR upper extremity/exp)
